# Synergistic and Antagonistic Drug Combinations Depend on Network Topology

**DOI:** 10.1371/journal.pone.0093960

**Published:** 2014-04-08

**Authors:** Ning Yin, Wenzhe Ma, Jianfeng Pei, Qi Ouyang, Chao Tang, Luhua Lai

**Affiliations:** 1 Center for Quantitative Biology, Peking University, Beijing, China; 2 BNLMS, State Key Laboratory for Structural Chemistry of Unstable and Stable Species, College of Chemistry and Molecular Engineering, Peking University, Beijing, China; 3 Peking-Tsinghua Center for Life Sciences, Peking University, Beijing, China; 4 School of Physics, Peking University, Beijing, China; 5 Department of Systems Biology, Harvard Medical School, Boston, Massachusetts, United States of America; Institute for Research in Biomedicine, Spain

## Abstract

Drug combinations may exhibit synergistic or antagonistic effects. Rational design of synergistic drug combinations remains a challenge despite active experimental and computational efforts. Because drugs manifest their action via their targets, the effects of drug combinations should depend on the interaction of their targets in a network manner. We therefore modeled the effects of drug combinations along with their targets interacting in a network, trying to elucidate the relationships between the network topology involving drug targets and drug combination effects. We used three-node enzymatic networks with various topologies and parameters to study two-drug combinations. These networks can be simplifications of more complex networks involving drug targets, or closely connected target networks themselves. We found that the effects of most of the combinations were not sensitive to parameter variation, indicating that drug combinational effects largely depend on network topology. We then identified and analyzed consistent synergistic or antagonistic drug combination motifs. Synergistic motifs encompass a diverse range of patterns, including both serial and parallel combinations, while antagonistic combinations are relatively less common and homogenous, mostly composed of a positive feedback loop and a downstream link. Overall our study indicated that designing novel synergistic drug combinations based on network topology could be promising, and the motifs we identified could be a useful catalog for rational drug combination design in enzymatic systems.

## Introduction

Drug combinations have been envisaged by many to be a promising approach to treat complex diseases such as cancer, inflammation and type 2 diabetes [1]–[3]. However, when used in combination, drugs interact in many unexpected ways and show a plethora of different outcomes [4]. Among these interactions, drug synergy and antagonism have attracted special attentions. Drug synergy, the combined boost of drug efficacy, is a highly pursued goal of combinational drug development [2]. Synergistic drug combinations have been shown to be highly efficacious and therapeutically more specific [5]. Drug antagonism, in contrast, is often undesirable, but could be useful in selecting against drug resistant mutations [6]. Despite active research into the mechanism of drug synergy or antagonism, the answer remains largely elusive. Experimentally, combinational high throughput screening [7]–[9] was devised to search for synergistic drug pairs in several systems. The low hit rate of drug synergy (generally less than 10%) stimulated many computational efforts to predict and quantify drug synergy. Li et al. [10] used an abstract network topology-based approach to predict drug synergy. Based on topological relationships between drug targets, they devised a synergy score to rank and select possible synergistic drug pairs. A chemical genomic approach was taken by Jansen et al. [11] to uncover antifungal synergies based on the assumption that drugs with similar chemogenomic profiles would more likely be synergistic. By surveying the existing synergistic drug pairs and their topological relations in biological networks, Zou et al. [12] suggested that synergistic drug target combinations tend to be in so called neighbor communities. Based on this concept they trained a support vector machine (SVM) classifier and successfully retrieved and experimentally confirmed several synergistic drug pairs. Noting the similarity between drug synergy and genetic interaction, Cokol et al. [13] suggested that gene pairs manifesting negative genetic interactions may be possible synergistic drug target pairs. The experiment they had conducted on yeast using this concept indeed showed enrichment of synergistic drug pairs, but many of these drug synergies were later found to be not related to the underlying genetic interactions. To fast simulate drug synergy on established molecular networks, Yan et al. [14] introduced a simplifying strategy for efficient calculation of scores representing synergistic interactions. Still, predicting drug synergy or antagonism is difficult. Seemingly synergistic combinations such as the antibiotic combination of DNA replication inhibitor and ribosome inhibitor are actually antagonistic [15], and context or sequence dependent synergy in some cases further complicated the problem. Thus it is of great interest to predict drug synergy or antagonism based on the topology of the drug target network.

Biological functions are carried out by many molecules interacting in a network-like manner. The network structure largely determines the dynamics of the interacting molecules, hence the function it can fulfill. Drug interactions may also be determined in such a manner, so that the structure of the biological network involving the drug targets under study may shed light into the way the drugs act [16]–[18] and interact [19], [20]. Indeed, an early study demonstrated theoretically that serial inhibition of an enzymatic chain can lead to drug synergy [21]. Fitzgerald et al. [2] examined different patterns of synergistic combination in several typical network contexts. Lehar et al. took a reverse approach and used the patterns of drug interactions outcomes to successfully infer the targets connectivity in metabolic pathways [19]. Though the relationship between network structures involving drug targets and patterns of drug interaction has been demonstrated in their studies, drug synergy/antagonism was thought to depend heavily on parameters [19]. We ask if the otherwise might be true, that network structure prevails over parameters in determining whether the drug combination is synergistic or not. In order to test this hypothesis, we comprehensively cataloged the drug combination outcomes in a model system and established the connection between the structure of target-related networks and drug synergy/antagonism it endows. There are many possible sources of drug interactions, but we focused exclusively on those combinations that do not involve pharmacokinetic interactions. Therefore, the drug interactions studied here arise from interactions of inhibited targets in the underlying network. Moreover, since we consider combined inhibition of targets, synergy exhibited by dual-inhibitors which inhibit two targets simultaneously will also be accounted by our models.

## Methods

### Modeling three-node enzymatic networks

**Figure 1:**
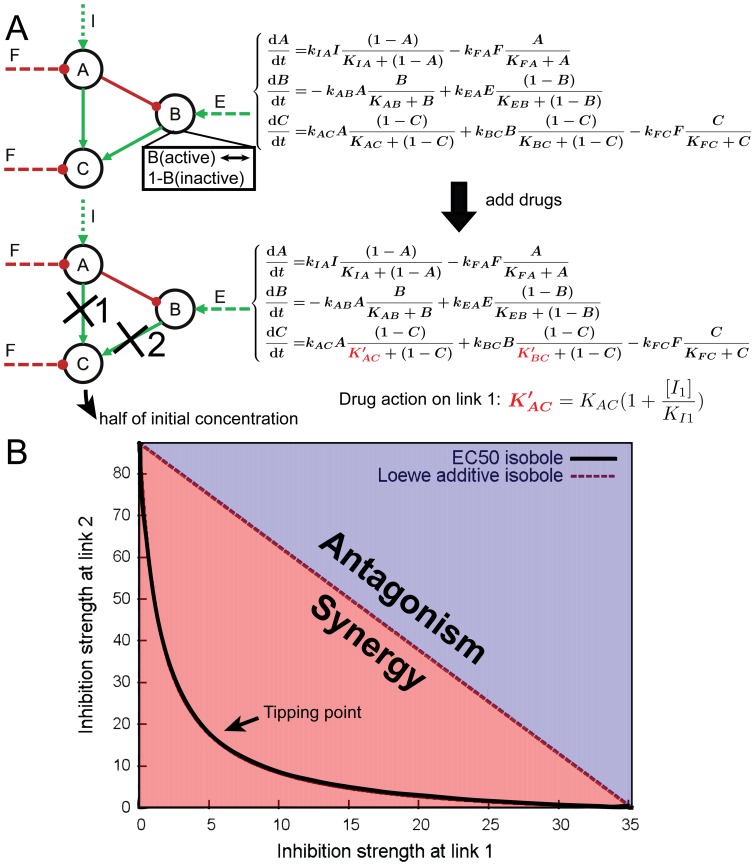
Modeling process to study drug combinations. (A) Illustration of the drug modelling process. An example enzymatic network with corresponding ODEs is shown. Solid links represent inter-node regulatory relationships, broken lines are background regulations. With the addition of drugs to chosen links (shown by crosses), the equations are modified by incorporating drugs as competitive inhibitors. (B) An example isobologram calculated from the combination of links 1 and 2 in (A). Points on isobloles represent dose combinations with the same efficacy. The black isobole (solid line) is concave, suggesting a synergistic interaction between the two links. The tipping point is the point where CI reaches minimum (or maximum for antagonistic cases). Inhibition strength is defined as [I]/Ki, i. e. the concentration of the inhibitor divided by its inhibition constant. The combination indices calculated from inhibition strengths are identical with those calculated with concentrations since K_I_'s cancel. The whole process depicted here was repeated for all (16,038) networks and 100,000 sampled parameter sets.

To extensively model drug action in diverse conditions and elucidate the connection between network topology and drug interactions, we chose to first study small networks which could be thought of as simplifications of disease related networks. A commonly used small-network formalism to investigate the topology-function relationship is the three-node enzymatic network studied by Ma et al. [22] and others [23], [24]. Because of the frequent use of enzymes as drug targets, we considered enzymatic network as a valid representation of a class of drug target related network. A three-node enzymatic network consists of three enzymes, each existing in active or inactive states. The concentration of each enzyme was 1 μM. Following a prescribed connective structure of the networks, the enzymes catalyze the reversible conversion of other enzymes from one state to the other, thus activate or deactivate them (Figure 1A). To ensure a positive steady state for each enzyme, if an enzyme receives only positive/negative regulations, a background negative/positive enzyme regulation F/E was added (E and F in equations in Figure 1A). All catalyzing reactions were modeled by Michaelis-Menten kinetics, and a background activating enzyme regulation I for node A serves as an input of the system. Concentrations of I and E/F were fixed at 1 μM and 0.5 μM, respectively. All free parameters in the system were generated by latin hypercube sampling [25], done logarithmic uniformly in a biological range of 0.001,10 μM for K_M_, and 0.1, 10 s^−1^ for k_cat_. We first solved the nonlinear equations with the nonlinear equation solver gsl_multiroot_fsolver_hybrids in GSL (GNU Scientific Library) [26] to obtain a steady state of the system, and then we performed linear stability analysis at the solution to select stable states. Linear stability analysis was performed following standard procedures: first the Jacobi matrix at the steady state was calculated; then the eigenvalues of the Jacobi matrix were computed. If all eigenvalues of the matrix had negative real parts, we considered the solution as a stable state and used it for further analysis.

### Modeling drug action on three-node enzymatic networks

One enzyme (node C in Figure 1A) from the three enzyme system was chosen as the output node, whose active form concentration in the stable steady state of the system was recorded to monitor the efficacy of drugs. Thus, if an inhibitor reduced the active form concentration of the output node by a certain percentage (50% reduction used in the current study) from the drug-free value, it would be considered as a candidate drug and the reaction (a specific link in the network) it targeted would be a candidate drug target. After identifying all candidate drug targets, their combinations were studied by computational enumeration. We used the concepts of Loewe synergy [27] and combination index (CI) [28] to distinguish between drug synergy and antagonism. CI is defined as follows: the denominators are the EC_50_'s of the drugs acting alone, whereas the numerators are the concentrations of the drugs in a combination that exert the same 50% reduction effect.
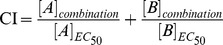



For each drug combination, an EC_50_-isobole was computed (Figure 1B) and CI was calculated throughout the concentration range. A CI consistently less than 1 (a downward concave isobole) represents drug synergy; a CI greater than 1 (an upward concave isobole) indicates drug antagonism. Thresholds of 1.01 and 0.99 were used in the computation. Drug pairs with CIs between 0.99 and 1.01 were classified as Loewe additive. We also tested larger margins (such as 0.9 and 1.1), and the results were qualitatively similar. The whole process is illustrated in Figure 1.

### Sampling all possible network topologies for drug interaction patterns

To obtain a complete catalog of patterns of drug synergy/antagonism in our model system, we enumerated all possible network interaction patterns (each node could have three possible links to itself and other two nodes, and the links could be activation or deactivation, generating a total of 3^9^ networks. Eliminating networks with no connection from input (A) to output (C) leaves 16,038 networks) for the three-node enzyme network following Ma et al. [22] For each of these networks, the complete Michaelis-Menten reaction kinetics was written as a set of ordinary differential equations (ODEs). After evaluating a stable steady state of the system by nonlinear equation solving and linear stability analysis, the process of drug action modeling described above was conducted. For each possible network topology, we ran a total of 100,000 simulations, each with a random parameter set generated by latin hypercube sampling. Drug interactions behaving consistently under various parameterizing conditions as synergistic or antagonistic were selected, clustered by network Hamming distance (number of differing links between two networks) and further analyzed. The values of CIs at the tipping point of the isoboles (Figure 1B), which we refer to as CI_t_'s, were also recorded as representations of the extent of synergy or antagonism.

## Results

### Drug synergy/antagonism is a property largely determined by network topology

**Figure 2:**
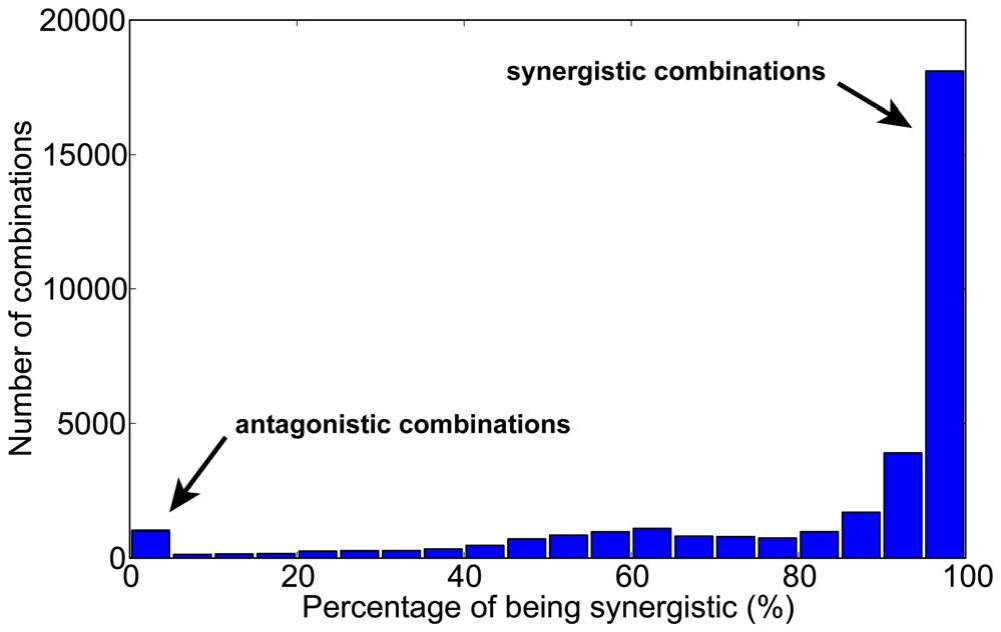
Distribution of percentage of synergistic cases under various parameter sets for all combinations studied. Consistently synergistic and antagonistic combinations are marked, showing their stark contrast in number.

**Figure 3:**
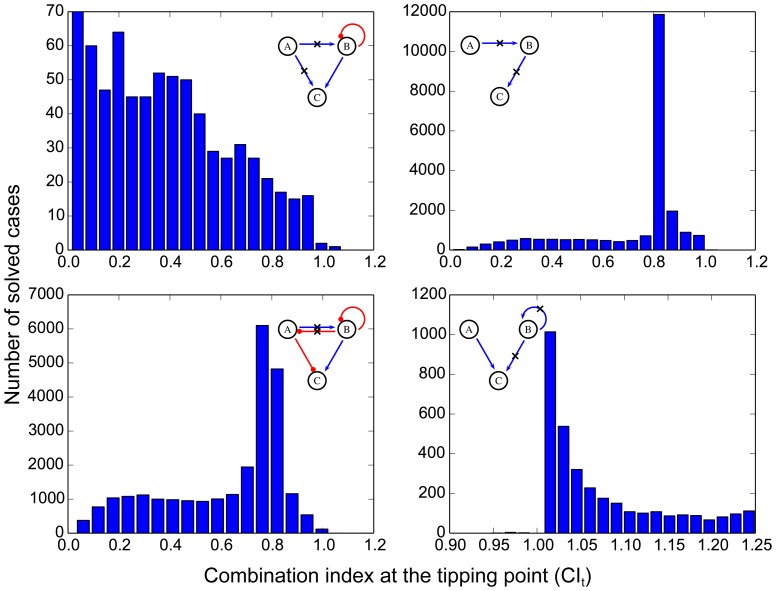
Distribution of CI_t_ values for several combinations. The combinations are shown in insets and the targets inhibited marked by crosses. These combinations are all highly consistent in showing either synergy or antagonism under 90% of parameterizing conditions.

We modeled patterns of possible drug combinations in all possible three-node enzymatic network topologies, using pre-sampled 100,000 parameter sets (Work flow summarized in Figure 1, detailed in Methods). Because of the complex dynamics in many networks, and the requirements that the inhibition strength ([I]/K_I_) falls within physiological range, we could only calculate a handful of closed isoboles in many drug combination cases. We hence collected drug combinations for which we have solved more than 100 cases and exclusively studied them. In total, there are 33,798 cases of drug combinations in various network structures that are solved successfully for more than 100 parameter sets. We next examined how these drug combinations behave under various parameterizing conditions. The relative inertness of drug interaction patterns to parameter changes was observed. For most combinations studied in our calculations, whether the drug interaction was synergistic or antagonistic, was robustly consistent under most parameterizing conditions. The distribution of percentage of synergy in all combinations is shown in Figure 2. It could be seen that more than 18,000 drug combinations were synergistic in more than 95% of all their solved cases. Therefore, if we specified a specific target combination in an enzymatic network, the pattern of interaction of drugs targeting them would be largely determined by the network structure. This was in contrast to the previous idea that one could calculate whatever values of CI by varying the parameters of a specified network [19]. Indeed, CI would vary under different parameterizing conditions, but the qualitative nature of the combination, i.e. drug synergy or antagonism, remains relatively inert. To illustrate this point, we plotted the distribution of CI_t_'s for several combinations in Figure 3. For each of the combinations shown, although the CI_t_ values vary over a wide range, they fall consistently in either the synergistic range (<1) or antagonistic range (>1). Overall, we believe that this is a strong evidence for our hypothesis that the qualitative nature of drug interaction was largely determined by the topology of the network involving the drug targets, while parameters conferring only a minor influence.

### Synergistic combinations are abundant in our model

**Figure 4:**
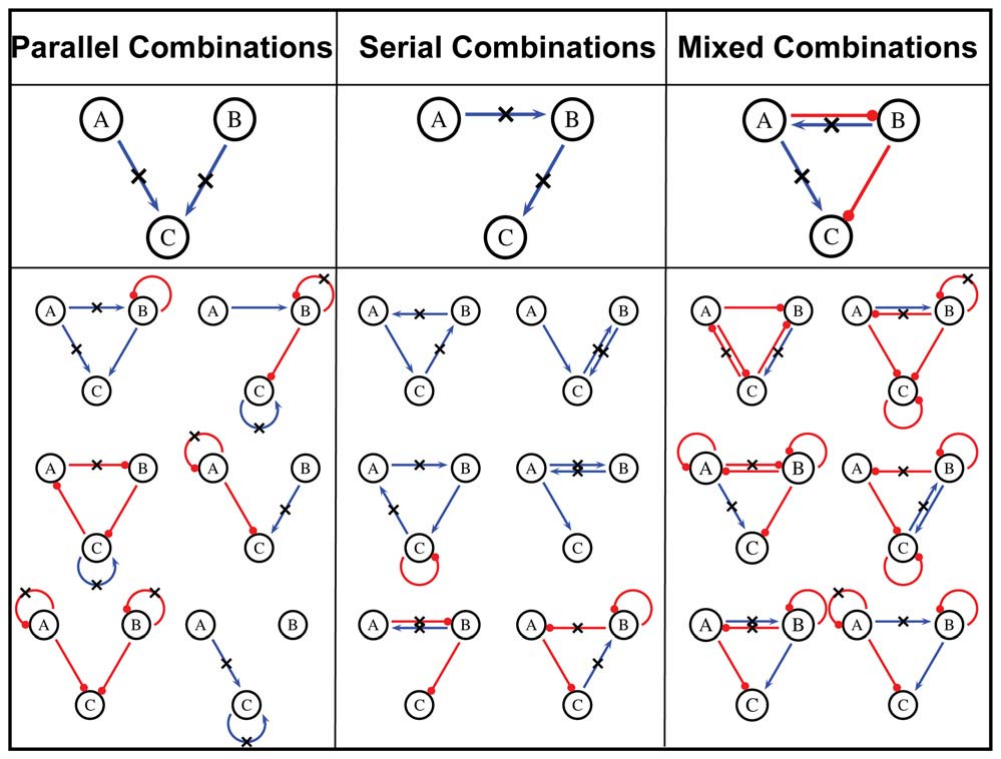
List of basic motifs that could result in drug synergy. Red dotted arrows indicate inhibitory actions, while blue arrows indicate activations. The targeting links of the drug combinations are marked by crosses in each network.

Figure 2 shows the distribution of percentage of solved cases being synergistic for all 33,798 drug combinations we have studied. It could be seen that more than 18,000 drug combinations are synergistic for more than 95% of the cases, whereas only around 1,000 combinations are consistently antagonistic. We selected those motifs (8241 networks) with more than 500 solved cases in which 99% or more are synergistic. These motifs were clustered according to the network structure. From visual inspection of the representative networks in each cluster, we identified the common skeleton shared by cluster members. These ‘synergistic motifs’ were basic building blocks that could lead to drug synergy. We then classified these motifs into three broad categories: serial, parallel and mixed serial-parallel combinations. As shown in Figure 4, each category includes diverse network topologies. It should be noted that these structures are themselves ‘motifs’, i.e. adding additional links or feedbacks usually do not alter synergistic interactions of the combinations.

### Quantitative comparison of parallel and serial drug combination patterns

**Figure 5:**
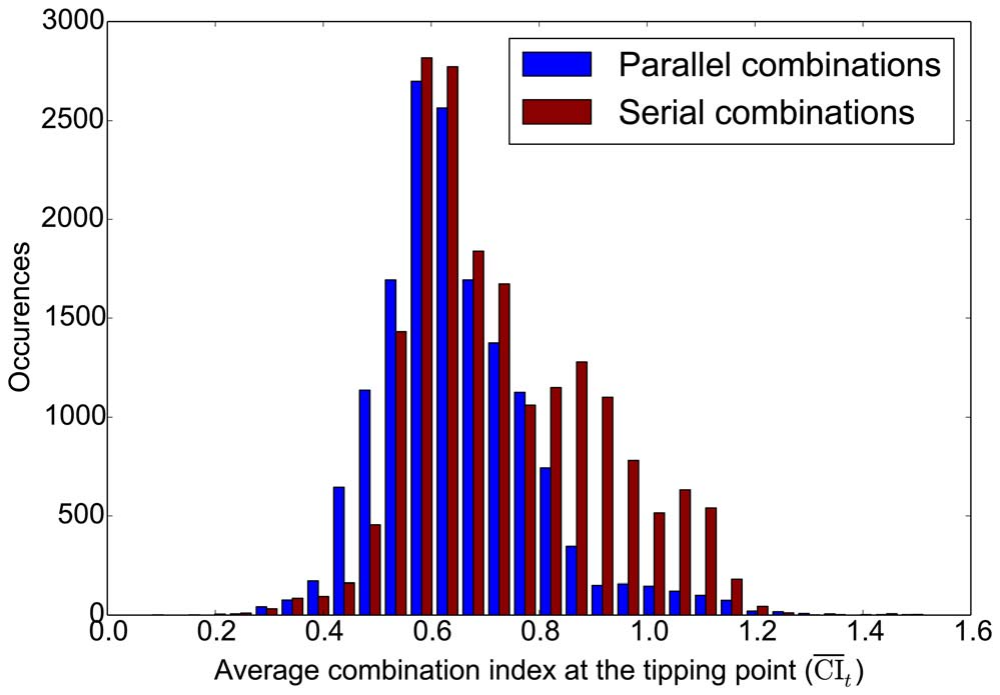
Comparison of distributions of average CI_t_'s for parallel and serial combinations. Definitions of parallel and serial combinations are presented in the main text.

As a quantitative measure, CI_t_ (Figure 1B) can be used to judge the extent of synergy or antagonism. When CI_t_ is less than 1, a smaller CI_t_ indicates a more concave bending of the isobole toward the lower concentration end, which in turn indicates a greater reduction of drug doses needed. Because CI_t_ varies with the parameters, we calculated average CI_t_ for each combination we studied as the average of all the CI_t_ 's in solved cases of the combination. To compare two major classes of combinational patterns, i.e. parallel and serial combinations, we separated all 33,798 combinations into these two groups. If the end node of the first target link served as the starting node of the second target link, we defined the combination of these two links as serial combination. All other combinations were treated as parallel. The distributions of the average CI_t_'s for all parallel and serial combinations are shown in Figure 5. Both distributions peak around 0.6, a mildly synergistic value. However, serial combinations in general have higher CI_t_ values than parallel combinations. Also, antagonistic combinations come from mainly serial combinations, which confirms the observations in the previous section. Therefore, parallel combinations generally show greater dose-reducing effects, and are less likely to be antagonistic.

### Antagonistic combinations in our model

**Figure 6:**
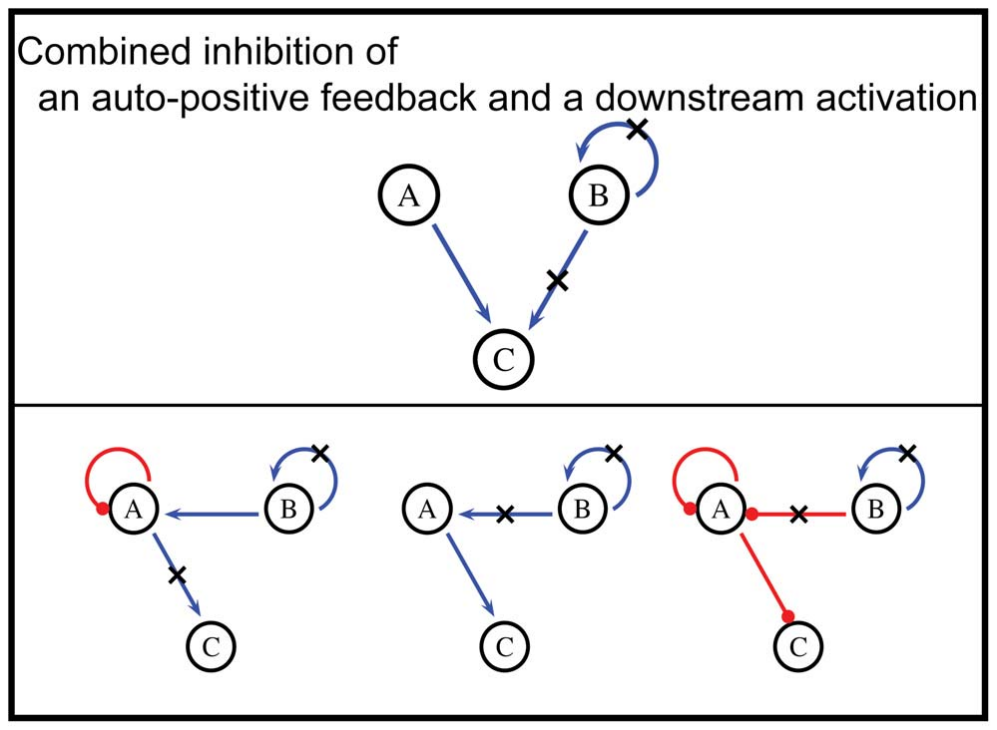
List of antagonistic combinations found in our study.

Since most drug combinations we found were synergistic, the less common antagonistic cases in our model deserve special attention. Similar to analysis of synergistic drug combinations, we selected those drug combinations (1044 combinations) with more than 100 solved cases showing antagonistic more than 90% of the time and clustered them. Only one major class of antagonistic interactions was identified (Figure 6). These antagonistic combinations all involved inhibition of a positive feedback on a node, and a downstream link originating from that node. On one hand, this suggests that it is relatively hard for two drugs to be antagonistic in small enzymatic networks; on the other hand, this motif serves as an interesting example of how antagonism could arise not from competing actions of two drugs, but from the specific network topology that underlies the drug targets. We investigated how this type of combinations leads to drug antagonism in the next section.

### Theoretical analysis of the origin of synergy and antagonism in typical motifs

We performed a theoretical analysis on the origins of synergy and antagonism in typical drug combination motifs. First, we derived a criterion for judging synergy or antagonism from the definition of Loewe synergy. Based on this criterion, we have dissected the source of synergy for the simplest cases of serial combination and parallel combination, as well as the basic motif of antagonistic combination (File S1). We noticed that single-drug dose response relationship is a key determinant of synergy/antagonism in our model system. In parallel/serial combinations, two hyperbolic single drug dose response relationships make a synergistic interaction easy to achieve. Inhibiting a positive feedback may produce a non-hyperbolic dose response relationship that is prone to antagonism. We have provided a detailed discussion in File S1.

## Discussion

### Synergy prevails in drug combinations targeting closely connected targets

**Figure 7:**
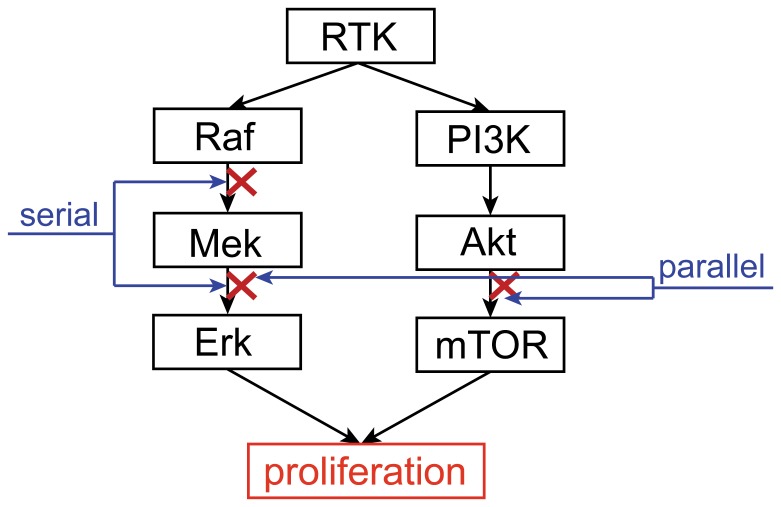
Basic structure of the growth factor signaling pathway, showing examples of serial and parallel combinations.

Our results demonstrated the high relevance of drug synergy in small scale enzymatic pathways. Previously Jansen et al. [11] reported enrichment of synergistic combinations in drug pairs with similar chemogenomic profiles, whose targets may be neighbors in the underlying biological networks. Our work provides a theoretical basis for how their approach is successful. Furthermore, the practice of targeted cancer therapy combinations have demonstrated the benefits of co-targeting closely related molecular targets, especially in the MAPK and the PI3K/Akt/mTOR pathways. For example, serial combinations such as Mek/Raf [29] and Akt/mTOR [30], or co-targeting closely related parallel pathway such as Mek/Akt [31] or Raf/mTOR [32] have all been shown to be synergistic (Fig. 7). Feedback and crosstalk abound in these two pathways, but consistent with our results, they generally do not alter the qualitative feature of the combination, so that most combinations of targets inside these pathways are synergistic. Thus, designing synergistic combinations targeting closely connected targets in many enzymatic pathway diseases is a promising strategy. Targeting unrelated drug targets in large networks often fail to show synergy, as exemplified by high throughput screening studies.

### Possible source for buffering antagonism

Yeh et al. [4], [20] categorized drug antagonism into two categories: antagonistic buffering and antagonistic suppression. In the case of antagonistic suppression, one drug suppresses the action of the other, producing a “hyper-antagonism” with a maximum combination index larger than 2. Well studied cases of drug antagonism are chiefly of this type, such as the combination of the antibiotics spiramycin and trimethoprim [15], and the combination of dexamethasone and paclitaxel for lung cancer chemotherapy [33]–[35]. We observed no cases of suppressive drug antagonism in our modeling study: all the antagonistic cases we identified were buffering antagonisms (one example shown in Fig. 3, lower right, where CI_t_ values falls between 1 and 2). Therefore, our results suggest that in antagonistic drug combinations, drugs do not necessarily jeopardize the action of each other as commonly thought. Instead, certain network topological arrangement of the drug targets, such as the motifs involving positive feedbacks in our findings, could naturally produce a buffering antagonism between the drugs. Whether such antagonism exists, and whether it contributes to clinically observed antagonism is another interesting topic for future investigations.

### Possible applications of the synergy/antagonistic catalogs

In contrast to previous ideas, we have shown that drug synergy or antagonism strongly depends on the underlying target-network topology for enzymatic systems. It is therefore useful to compile catalogs of synergistic or antagonistic combination motifs. Such catalogs can be exploited for rationally designing drug combinations, or multi-target drugs. For example, our calculations suggest that many motifs (Figure 4) are highly consistent in showing synergistic behaviors. If a disease-related biological network falls into one of the categories we found, then a combination could be safely proposed to be a synergistic combination. Also, the observation that simple serial and parallel combinations tend to be mostly synergistic could be a guideline that can be readily applied to many scenarios involving signal transduction pathways similar to those shown in Fig. 7. Previously, most synergistic combinations were proposed based on experience. Here we provide a rational and readily applicable approach toward synergistic drug combination design. We need to stress that our results come from calculations on enzymatic networks, and other types of biological networks still need to be further studied.

## Conclusion

This work describes a comprehensive study of the combined effects of drugs in three-node enzymatic networks. Drug synergy or antagonism was shown to be a property of target-related network topology. Several basic synergistic and antagonistic motifs were summarized and analyzed. Synergistic motifs could be classified into parallel, serial and mixed type combinations, whereas antagonistic combinations fall into one basic type involving positive feedbacks. Motifs described here can be used to design drug combinations in certain enzymatic contexts. Further work is warranted to clarify the effects of drug combinations on more complex biological networks.

## Supporting Information

File S1 **Theoretical analysis of basic synergy/antagonism motifs.**
(PDF)
