# Supplementary material for: Synergistic and Antagonistic Drug Combinations Depend on Network Topology
Source: PLoS One. 2014 Apr 8;9(4):e93960. doi: 10.1371/journal.pone.0093960 (PMC3979733; doi:10.1371/journal.pone.0093960)
Supplement: File S1 — Theoretical analysis of basic synergy/antagonism motifs. (PDF) [file pone.0093960.s001.pdf]

Supplementary document for Synergistic and antagonistic drug combinations depend on network topology

Ning Yin*, Wenzhe Ma||, Jianfeng Pei*, Qi Ouyang*¶‡, Chao Tang*¶‡, Luhua Lai*†‡

*Center for Quantitative Biology,
†BNLMS, State Key Laboratory for Structural Chemistry of Unstable and Stable Species, College of Chemistry and Molecular Engineering,
¶School of Physics,
‡Peking-Tsinghua Center for Life Sciences,
Peking University, Beijing, China.
||Department of Systems Biology,
Harvard Medical School, Boston, Massachusetts, U.S.A.

1 Derivation of a differential criterion for synergy judgement

The Loewe definition of synergy[1] essentially states that the Loewe additive response surface of two drugs, i.e. the dose-response function $f(x_1, x_2)$ * is a ruled surface[2]. If we allow the response functions of the two drugs to be different (as we do in this case), the additive surface is in general not developable. For any two single-drug dose-response functions $g(x_1)$ and $h(x_2)$, we can construct a Loewe additive surface by connecting the points $(x_1, 0, g(x_1))$ and $(0, x_2, h(x_2))$ where $g(x_1) = h(x_2)$ using straight line segments. If the true double response function $f(x_1, x_2)$ lies above this additive surface, these two drugs are synergistic, otherwise they are antagonistic. Therefore, if we cut the response surface by a vertical plane passing through one of the additive segment (Figure S1), the cross section of these two surface will reflect whether the two drugs are synergistic around the cutting point. A synergistic response surface should have a cross section like the one shown in Figure S2.

If the cross section is convex as in Figure S2, the drug combination will be synergistic. We could therefore derive a sufficient (not necessary) condition for synergy. The cross section could be written as a function of one variale x_1: $R(x_1) = f(x_1, h - kx_1)$, where h

*x_i's are the doses of the two drugs, while f is the efficacy of the two drugs used together at those doses.

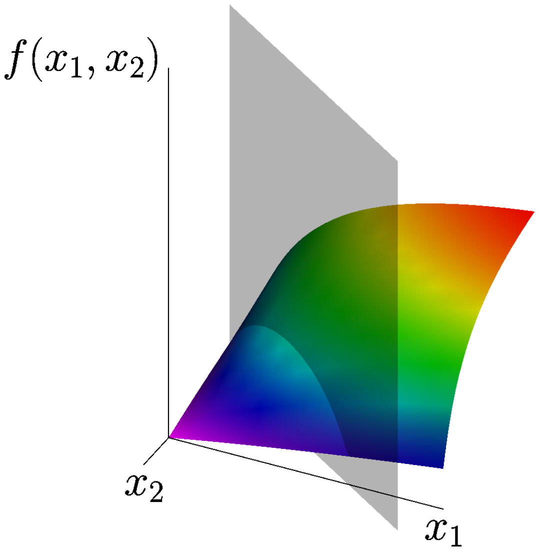

Figure S1: Cutting the response surface by a vertical plane

and k are constants. The convexity of $R(x_1)$ implies that

$$\frac{\mathrm{d}^2 R}{\mathrm{d}x_1^2} = f_1'^2 f_{22}'' + f_2' f_{11}'' - 2 f_1' f_2' f_{12}'' < 0$$

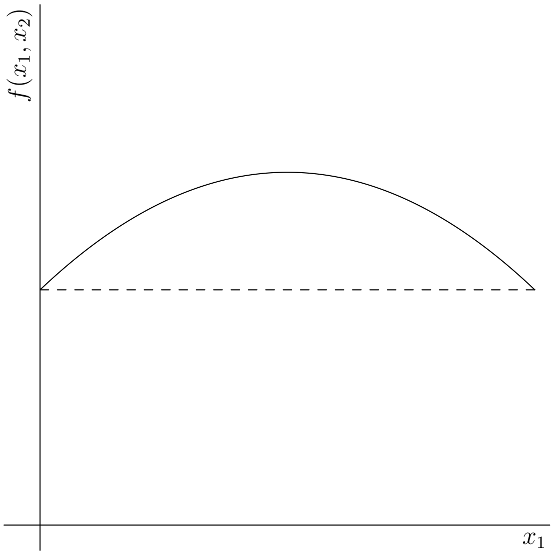

Figure S2: The cross section resulted from the cutting in Figure S1

We denote the lefthand side of the above equation as a discriminant Σ_f for synergy. In most cases we analyze below, we will employ linear approximations for Michaelis-Menten formalism.

2 Analysis of basic motifs for synergistic and antagonistic combinations

In this section we analyze some basic motifs of drug combinations we identified. We solve analytically the stable steady state of the three-node enzymatic network, and calculate the discriminant Σ_f for each case. Since we model the drug action as competitive inhibition, the drug effect is essentially the modification of the Michaelis constant K_M of the target link: $K'_\mathrm{M} = K_\mathrm{M}(1 + [I]/K_\mathrm{I})$. We represent the dose of the drug by $[I]/K_\mathrm{I}$ as in the main text, and it could be shown that if the dose-response function could be written as:

$$f(x_1, x_2) = f(K'_\mathrm{M1}(x_1), K'_\mathrm{M2}(x_2))$$

where $K'_\mathrm{Mi} = K_\mathrm{Mi}(1 + x_i)$, then

$$\Sigma_{f(x_1,x_2)} = K_\mathrm{M1}^2 K_\mathrm{M2}^2 \Sigma_{f(K'_\mathrm{M1},K'_\mathrm{M2})}$$

thus in the cases that follows, we only calculate $\Sigma_{f(K'_\mathrm{M1},K'_\mathrm{M2})}$, since it has the same sign as the original discriminant.

2.1 Serial combination

For a pure serial combination of two inhibitors (Figure S3), we have

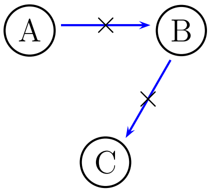

Figure S3: Basic motif of serial combination

$$\begin{cases} \dfrac{\mathrm{d}[B]}{\mathrm{d}t} = k_1[A](1 - [B]) - k_3 F[B] \\ \dfrac{\mathrm{d}[C]}{\mathrm{d}t} = k_2[B](1 - [C]) - k_4 F[C] \end{cases}$$

Where $k_1 = k_\mathrm{cat1}/K_\mathrm{M1}$, $k_2 = k_\mathrm{cat2}/K_\mathrm{M2}$ The solution of steady state $[C]$ is

$$[C] = \frac{k_1 k_2 [A]}{k_1(k_2 + k_4 F)[A] + k_3 k_4 F^2}$$

Which can be converted into

$$[C] = \frac{a}{y(bx + c) + a}$$

where $x = K_{\mathrm{M1}}$, $y = K_{\mathrm{M2}}$ and

$$\begin{cases} a = k_{\mathrm{cat1}} k_{\mathrm{cat2}} [A] \\ b = k_3 k_4 F^2 \\ c = k_{\mathrm{cat1}} k_4 F [A] \end{cases}$$

hence the response function can be written as

$$f(x, y) = [C]_0 - [C] = [C]_0 - \frac{a}{y(bx + c) + a}$$

Note that both f_{xx} and f_{yy} are < 0, indicating hyperbolic dose-response relationships:

$$f_{xx} = -\frac{2ab^2y^2}{(a + (c + bx)y)^3}$$

$$f_{yy} = -\frac{2a(c + bx)^2}{(a + (c + bx)y)^3}$$

Although the sign of f_{xy} is indeterminate, it could be shown that

$$\Sigma_f = -\frac{2a^3b^2(c + bx)y}{(a + (c + bx)y)^6} < 0$$

Therefore synergy is guaranteed.

2.2 Parallel combination

For parallel combination (Figure S4) only one equation is involved:

$$\frac{\mathrm{d}[C]}{\mathrm{d}t} = k_1[A](1 - [C]) + k_2[B](1 - [C]) - k_3 F [C]$$

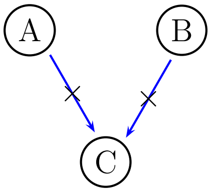

Figure S4: Basic motif of parallel combination

the steady state of the system is

$$[C] = \frac{k_1[A] + k_2[B]}{k_1[A] + k_2[B] + k_3 F}$$

again we write the response function in a simplified form:

$$f(x,y) = [C]_0 - \frac{ax+by}{ax+by+cxy}$$

where again $x = K_{\mathrm{M1}}$, $y = K_{\mathrm{M2}}$ and

$$\begin{cases} a = k_{\mathrm{cat2}}[B] \\ b = k_{\mathrm{cat1}}[A] \\ c = k_3 F \end{cases}$$

Again, f_{xx} and f_{yy} are both < 0:

$$f_{xx} = -\frac{2bcy^2(a+cy)}{(ax+(b+cx)y)^3}$$

$$f_{yy} = -\frac{2acx^2(b+cx)}{(ax+(b+cx)y)^3}$$

moreover, $f_{xy} > 0$ for the parallel combination:

$$f_{xy} = \frac{2abcxy}{(ax+(b+cx)y)^3}$$

which makes a positive Σ_f guaranteed. Indeed we have

$$\Sigma_f = -\frac{2abc^3x^2y^2(ax+by)}{(ax+(b+cx)y)^6} < 0$$

2.3 Positive feedback & its downstream link

Here we consider here the basic antagonistic motif we found: combination of an inhibition of an positive feedback and its downstream activation link (Figure S5)

$$\begin{cases} \frac{\mathrm{d}[B]}{\mathrm{d}t} = \frac{k_1[B](1-[B])}{K_1+1-[B]} - \frac{k_3F[B]}{K_3+[B]} \\ \frac{\mathrm{d}[C]}{\mathrm{d}t} = k_2[B](1-[C]) - k_4F[C] \end{cases}$$

Here for $[B]$ we kept the original Michaelis-Menten form to preserve the bistability of the system, and use the high steady state for our response function:

$$[B]_h = \frac{1}{2}(b + \sqrt{b^2 + 4(c - dK_1)})$$

thus the steady state concentration of C would be

$$[C] = \frac{b + \sqrt{b^2+4(c-dx)}}{b+\sqrt{b^2+4(c-dx)}+2ay}$$

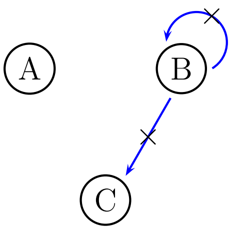

Figure S5: Basic motif of antagonistic combinations we identified. (Here we ignored the links from the input node A.)

and

$$f(x,y) = [C]_0 - [C] = [C]_0 - \frac{b + \sqrt{b^2 + 4(c - dx)}}{b + \sqrt{b^2 + 4(c - dx)} + 2ay}$$

here again $x = K_{\text{M1}}$, $y = K_{\text{M2}}$ and

$$\begin{cases} a = k_4 F / k_{\text{cat2}} \\ b = \dfrac{k_1 - k_1 K_3 + k_3 F}{k_1} \\ c = \dfrac{k_1 K_3 - k_3 F}{k_1} \\ d = \dfrac{k_3 F}{k_1} \end{cases}$$

We note that $f_{xx} > 0$ (Inhibition of the positive feedback) in this case:

$$f_{xx} = \frac{8ad^2y(b + 3\sqrt{b^2 + 4(c - dx)} + 2ay)}{(b^2 + 4(c - dx))^{3/2}(b + \sqrt{b^2 + 4(c - dx)} + 2ay)^3}$$

although f_{yy} remains negative:

$$f_{yy} = -\frac{8a^2(b + \sqrt{b^2 + 4(c - dx)})}{(b + \sqrt{b^2 + 4(c - dx)} + 2ay)^3}$$

And Σ_f is

$$\Sigma_f = \frac{128a^3d^2y(b^3 + b^2(\sqrt{b^2 + 4(c - dx)} + ay))}{(b^2 + 4(c - dx))^{3/2}(b + \sqrt{b^2 + 4(c - dx)} + 2ay)^7)} + \frac{128a^3d^2y((c - dx)(\sqrt{b^2 + 4(c - dx)} + 2ay) + b(3(c - dx) + ay\sqrt{b^2 + 4(c - dx)}))}{(b^2 + 4(c - dx))^{3/2}(b + \sqrt{b^2 + 4(c - dx)} + 2ay)^7)}$$

Where a simple criterion $c - dx > 0$ would make it positive. The criterion is achieved when node B is bistable with two positive steady states. This requires that $c < 1, \quad x = K_{\text{M1}} < \frac{c}{d}$.

3 Summary

The structure of the discriminant is actually quite self-explanatory:

$$\Sigma_f = f_1'^2 f_{22}'' + f_2' f_{11}'' - 2 f_1' f_2' f_{12}''$$

The first two terms describe 'self-interactions', while the last term describes the 'cross interaction'. When the 'cross interaction' exceeds the 'self-interaction', synergy emerges. Therefore, the 'self-interactions' play an important role in determining the combinational response. For the serial combination and the parallel combination we analyzed above, the single-drug dose response functions are hyperbolic in shape, showing negative 'self-interaction', thus making them easy be behave synergistically. However, the inhibition of the positive feedback sometimes produce a potive 'self-interaction' term (when the system resides in the higher state of the bistable states), thus making the combination more prone to antagonistic behaviour. We do not know whether this kind of behaviour exists real-life cases, but it may be a reason behind some experimentally observed antagonistic behaviours.

If we model the reactions between the three enzymes with a Hill function (with Hill coefficient 2, i.e. change $\frac{x}{K+x}$ to $\frac{x^2}{K^2+x^2}$) rather than Michaelis-Menten formalism, the single-drug dose-responses are often sigmoidal. Sigmoidal dose-response functions differ from hyperbolic dose-response functions in that they have positive 'self-interaction' parts and negative 'self-interaction' parts. Presumably, under this situation the synergistic cases would be reduced, and most cases might tend to be additive or antagonistic. This is exactly what we have found (Figure S6). Therefore, adding more 'nonlinearity' to the system actually decreases the incidences of synergy. This is another possible reason for the experimentally observed rarity of synergistic combinations.

References

[1] W. R. Greco, G. Bravo, and J. C. Parsons. The search for synergy: a critical review from a response surface perspective. *Pharmacol. Rev.*, 47(2):331–385, Jun 1995.

[2] Manfredo P. Do-Carmo. *Differential Geometry of Curves and Surfaces.* Prentice Hall, first edition, February 1976. ISBN 0132125897.

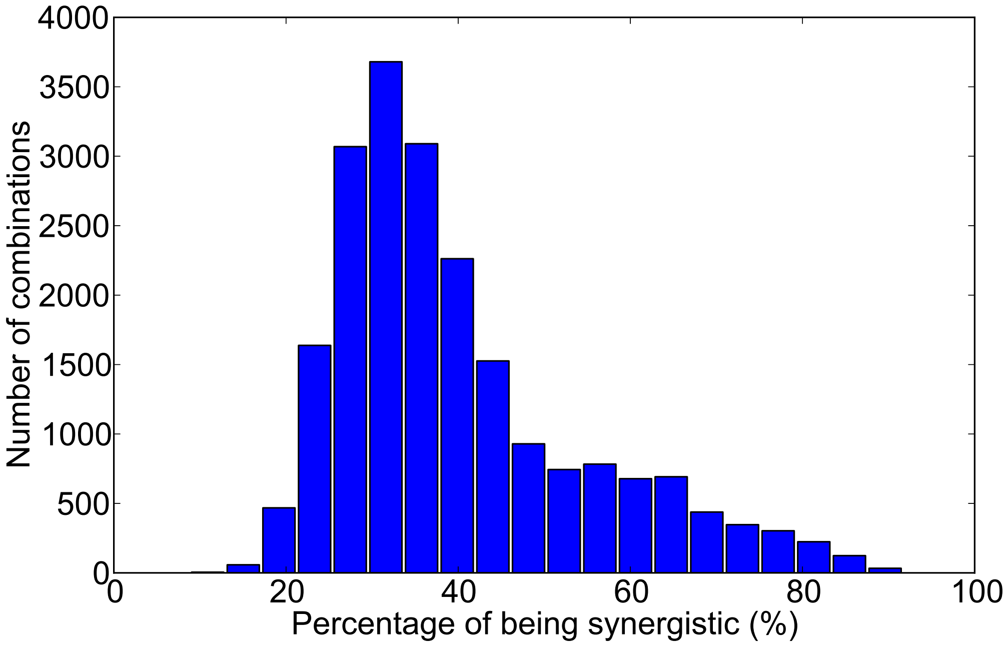

Figure S6: Distribution of percentage of synergistic cases under various parameter sets using Hill-type kinetics (Compare with Figure 2 in main text)
